# A short anogenital distance on MRI is a marker of endometriosis

**DOI:** 10.1093/hropen/hoab003

**Published:** 2021-02-17

**Authors:** A Crestani, C Abdel Wahab, A Arfi, S Ploteau, K Kolanska, M Breban, S Bendifallah, C Ferrier, E Darai

**Affiliations:** 1 Department of Gynaecology and Obstetrics, Tenon University Hospital, Assistance Publique des Hôpitaux de Paris (AP-HP), Sorbonne University, Paris, France; 2 Department of Radiology, Tenon University Hospital, Assistance Publique des Hôpitaux de Paris (AP-HP), Sorbonne University, Paris, France; 3 Service de Gynécologie-Obstétrique, CIC FEA, Hôpital Mère Enfant, CHU Hôtel Dieu, Nantes, France; 4 UMRS 938, Centre de recherche Saint Antoine, Faculté de Médecine Sorbonne Université, Paris, France; 5 INSERM UMR_S_707, ‘Epidemiology, Information Systems, Modeling’, University Pierre and Marie Curie, Paris, France; 6 Groupe de recherche clinique (GRC-6), Centre Expert En Endométriose (C3E), Assistance publique des hôpitaux de Paris, hôpital Tenon, Sorbonne Université, Paris, France

**Keywords:** anogenital distance, endometriosis, MRI, optimal cut-off, fertility, endocrine disruptor

## Abstract

**STUDY QUESTION:**

Could the anogenital distance (AGD) as assessed by MRI (MRI-AGD) be a diagnostic tool for endometriosis?

**SUMMARY ANSWER:**

A short MRI-AGD is a strong diagnostic marker of endometriosis.

**WHAT IS KNOWN ALREADY:**

A short clinically assessed AGD (C-AGD) is associated with the presence of endometriosis.

**STUDY DESIGN, SIZE, DURATION:**

This study is a re-analysis of previously published data from a case–control study.

**PARTICIPANTS/MATERIALS, SETTING, METHODS:**

Women undergoing pelvic surgery from January 2018 to June 2019 and who had a preoperative pelvic MRI were included. C-AGD was measured at the beginning of the surgery by a different operator who was unaware of the endometriosis status. MRI-AGD was measured retrospectively by a senior radiologist who was blinded to the final diagnosis. Two measurements were made: from the posterior wall of the clitoris to the anterior edge of the anal canal (MRI-AGD-AC), and from the posterior wall of the vagina to the anterior edge of the anal canal (MRI-AGD-AF).

**MAIN RESULTS AND THE ROLE OF CHANCE:**

The study compared MRI-AGD of 67 women with endometriosis to 31 without endometriosis (controls). Average MRI-AGD-AF measurements were 13.3 mm (±3.9) and 21.2 mm (±5.4) in the endometriosis and non-endometriosis groups, respectively (*P* < 10^−5^). Average MRI-AGD-AC measurements were 40.4 mm (±7.3) and 51.1 mm (±8.6) for the endometriosis and non-endometriosis groups, respectively (*P* < 10^−5^). There was no difference of MRI-AGD in women with and without endometrioma (*P* = 0.21), or digestive involvement (*P* = 0.26). Moreover, MRI-AGD values were independent of the revised score of the American Society of Reproductive Medicine and the Enzian score. The diagnosis of endometriosis was negatively associated with both the MRI-AGD-AF (*β* = −7.79, 95% CI (−9.88; −5.71), *P* < 0.001) and MRI-AGD-AC (*β* = −9.51 mm, 95% CI (−12.7; 6.24), *P* < 0.001) in multivariable analysis. Age (*β* = +0.31 mm, 95% CI (0.09; 0.53), *P* = 0.006) and BMI (*β* = +0.44 mm, 95% CI (0.17; 0.72), *P* = 0.001) were positively associated with the MRI-AGD-AC measurements in multivariable analysis. MRI-AGD-AF had an AUC of 0.869 (95% CI (0.79; 0.95)) and outperformed C-AGD. Using an optimal cut-off of 20 mm for MRI-AGD-AF, a sensitivity of 97.01% and a specificity of 70.97% were noted.

**LIMITATIONS, REASONS FOR CAUTION:**

This was a retrospective analysis and no adolescents had been included.

**WIDER IMPLICATIONS OF THE FINDINGS:**

This study is consistent with previous works associating a short C-AGD with endometriosis and the absence of correlation with the disease phenotype. MRI-AGD is more accurate than C-AGD in this setting and could be evaluated in the MRI examination of patients with suspected endometriosis.

**STUDY FUNDING/COMPETING INTEREST(S):**

N/A.

**TRIAL REGISTRATION NUMBER:**

The protocol was approved by the ‘Groupe Nantais d’Ethique dans le Domaine de la Santé’ and registered under reference 02651077.

**WHAT DOES THIS MEAN FOR PATIENTS?**

Endometriosis is a difficult disease to diagnose. Previous studies have indicated that the size of the outer part of the female genitals (vulva) is affected by exposure to chemicals that disrupt hormones (such as oestrogen) in the body: these chemicals may also be responsible for the development of endometriosis. The distance between the anus and the genitalia (anogenital distance (AGD)) is influenced by exposure to hormones and it may be shorter in women with confirmed endometriosis. Therefore, AGD may be a non-invasive marker for the diagnosis of endometriosis.

In a previous study, we demonstrated that directly measured vulva size (clinical measurement, using a ruler) was associated with the diagnosis of endometriosis. However, a medical imaging technique used in radiology (called MRI) to form pictures of the anatomy of the body has not been used to measure the AGD. We wanted to find out if using MRI to measure AGD would help in diagnosis of endometriosis.

In this study, by doing a re-analysis of information we had collected earlier from measuring vulvar size on MRI and in the operating room during surgery, we found that MRI measurement of AGD was more effective than clinical measurement in supporting the diagnosis of endometriosis. Our results suggest that measuring AGD using MRI could be a helpful non-invasive approach to diagnosing endometriosis.

## Introduction

Endometriosis is an oestrogen-dependent disease that affects up to 10% of women of reproductive age. The diagnosis of endometriosis remains challenging because of the heterogeneity of clinical presentation, the inconsistency of clinical signs, and the sub-optimal accuracy and operator-dependent aspect of radiological examinations (Nisenblat *et al.*, 2016c; Zondervan *et al.*, 2020). Women often have to wait for years after the onset of clinical signs before diagnosis (Nnoaham *et al.*, 2011; Bontempo and Mikesell, 2020; Ghai *et al.*, 2020).

In a meta-analysis, Nisenblat *et al.* (2016c) found that neither transvaginal ultrasound (TVUS) nor MRI can be used as a replacement or even triage, test to detect any type of pelvic endometriosis. This is particularly true for early stages of the disease, which are often restricted to the peritoneum (Bazot *et al.*, 2009; Nisenblat *et al.*, 2016a; Bazot and Daraï, 2017). Although TVUS and MRI have high accuracies for diagnosing endometrioma and colorectal endometriosis, the sensitivities of these techniques for some deep endometriosis (DE) locations, such as uterosacral endometriosis, the most common of DE lesions, are 0.64 and 0.81, respectively. Imaging techniques also have low accuracy for diagnosing superficial endometriosis (Bazot *et al.*, 2009; Nisenblat *et al.*, 2016a; Bazot and Daraï, 2017).

In this setting, effective diagnostic tools for endometriosis would be helpful for physicians. For decades, numerous potential non-invasive diagnostic biomarkers (blood, urine, endometrial) have been explored (Fassbender *et al.*, 2015) though none have been shown to be effective to date (Liu *et al.*, 2015; Gupta *et al.*, 2016; Nisenblat *et al.*, 2016b; Ahn *et al.*, 2017). According to a Cochrane review, even when associated with clinical examination or TVUS, combinations of different biomarkers failed to improve the diagnosis of endometriosis (Nisenblat *et al.*, 2016c).

Recent studies have focused on the relevance of the AGD for the diagnosis of endometriosis. The AGD in foetuses has been shown to be a marker of intra-uterine hormonal exposure (Dean and Sharpe, 2013; Swan *et al.*, 2015). Furthermore, a recent meta-analysis has established an epidemiological link between endometriosis and exposure to endocrine disruptors (Cano-Sancho *et al.*, 2019). A shorter AGD in patients with endometriomas and rectal DE was first described in Spain (Mendiola *et al.*, 2016). Then, in a cohort of women undergoing laparoscopy, we described a shorter AGD in women with histologically confirmed endometriosis, regardless of the stage and severity of the disease (Crestani *et al.*, 2020). Thus, AGD seems to be an interesting diagnostic marker. However, to date, MRI has not been used to measure the AGD.

This study is a re-analysis of our previously published data (Crestani *et al.*, 2020). Our aim was to compare the AGD as measured by MRI (MRI-AGD) in patients with endometriosis diagnosed on laparoscopic and histological findings with a non-endometriosis group, and thus to explore the diagnostic value of MRI-AGD. We also compared MRI-AGD values with clinical measurements of AGD (C-AGD) in patients with endometriosis.

## Materials and methods

### Study population

We carried out a case–control study using data collected from January 2018 to June 2019 in our tertiary care centre (Tenon University Hospital, Paris, France). The initial cohort, which included 98 patients with surgically and histologically proven endometriosis and 70 patients without endometriosis, has been described in a previous study (Crestani *et al.*, 2020).

Patients over 18 years old who underwent scheduled or emergency pelvic surgery in the gynaecological department, with an available pelvic MRI allowing an MRI-AGD measurement, were included. Pregnant and menopausal women were excluded from the study.

The following parameters were extracted from the dataset: age at surgery, parity, history of vaginal delivery, BMI, smoking status, presence of endometrioma, superficial endometriosis and DE on imaging (MRI and TVUS), history of infertility before surgery, symptoms, previous surgery for endometriosis, type and route of surgery (laparoscopy or laparotomy) and surgical findings (localization and severity of the endometriosis). Before the beginning of the surgery, a C-AGD measurement was made in all the women, as previously described (Crestani *et al.*, 2020).

### C-AGD and MRI-AGD measurement

C-AGD was measured before the beginning of the surgery in the lithotomy position and thighs at a 45° angle to the examination table. Two measurements were performed using a centimetre ruler with millimetre accuracy: from the clitoral surface to the anus (C-AGD-AC), and from the posterior fourchette to the anus (C-AGD-AF). The measurements were not carried out by the surgeon but by a second operator unaware of the patient’s pathology. The surgeon was blinded to the AGD-AC and AGD-AF values.

MRI-AGD was measured on a sagittal T2 weighted-imaging sequence by a radiologist who was blinded to the final surgical and histological diagnosis. All MRI images were obtained using a 1.5 or 3 T system (General Electric Medical Systems, Waukesha, WI, USA) with a dedicated pelvic 12 element phased-array coil and reviewed on a Picture Archiving and Communication System (PACS) (Carestream Health, Rochester, NY, USA).

**Figure 1.**
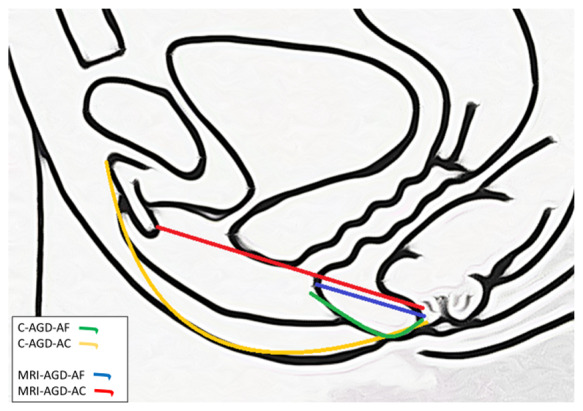
**Measurements of anogenital distance.** Anogenital distance (AGD) was measured clinically (C) using a ruler, or after MRI. C-AGD-AC: from the clitoral surface to the anus; C-AGD-AF: from the posterior fourchette to the anus; MRI-AGD-AC: from the posterior wall of the clitoris to the anterior edge of the anal canal; MRI-AGD-AF: from the posterior wall of the vagina to the anterior edge of the anal canal.

Two measurements were performed using straight lines with millimetre accuracy: from the posterior wall of the clitoris to the anterior edge of the anal canal (MRI-AGD-AC), and from the posterior wall of the vagina to the anterior edge of the anal canal (MRI-AGD-AF) (Fig. 1 and Supplementary Fig. S1).

### Statistical analysis

Statistical analyses were carried out using Stata/IC 14.0 (StataCorp LLC4905 Lakeway Drive, College Station, TX, USA), with significance value set at *P* = 0.05. Data are presented as mean ± SD for continuous variables or *n* (%) for categorical variables, where appropriate. To compare the variables across groups, the Student’s *t*-test and ANOVA were used for normally distributed data, the Mann–Whitney *U* test for non-parametric data and the Chi-square test for categorical data.

Simple and multiple linear regression models were conducted to identify a correlation between individual characteristics and both MRI-AGD-AC and MRI-AGD-AF. Variables that correlated with the MRI-AGD (with a *P*-value < 0.15) in the simple linear regression were incorporated in the multiple linear regression.

The AUC for the receiver operating characteristic (ROC) curve measured the ability to discriminate the presence of endometriosis, with an AUC of 0.5 indicating no discrimination and a value of 1, perfect discrimination.

We estimated the optimal cut-off to correlate both AGD and presence of endometriosis. The optimal cut-off was determined by a minimum *P*-value approach. This involved dichotomizing the MRI-AGD into dummy variables with a cut-off every 5 mm. The cut-off with the lowest *P*-value was chosen as the optimal cut-off for this variable.

### Ethical approval

The protocol was approved by the ‘Groupe Nantais d’Ethique dans le Domaine de la Santé’ and registered under reference 02651077.

## Results

### Epidemiological characteristics of the population

**Figure 2.**
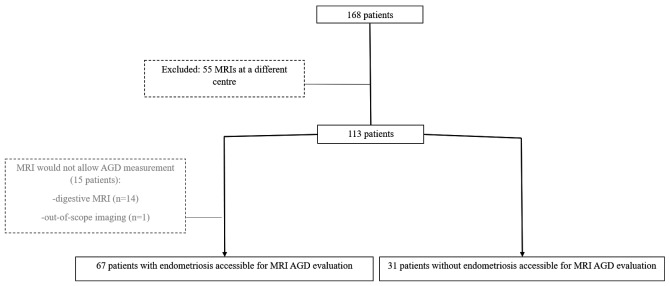
**Flow chart of participants in the study of endometriosis diagnosis by measuring AGD on MRI.** AGD, anogenital distance.

In the initial cohort, 98 patients underwent a pelvic MRI in our centre. Sixteen were excluded because the MRI was not suitable for MRI-AGD measurement: 15 were digestive MRI, and one a pelvic MRI but with the AGD out-of-scope. Thus 67 cases were included in the endometriosis group and compared with 31 controls in the non-endometriosis group (Fig. 2). Characteristics of the population are detailed in Table I.

**Table I:** Characteristics of the populations.

Patients	Endometriosis group (n = 67)	Non-endometriosis group (n = 31)	*P*-value
Age (years), mean (SD)	33.1 (6.5)	36.5 (10.4)	0.05
BMI (kg⋅m^−2^), mean (SD)	24.5 (5.2)	25.6 (6.0)	0.40
Parity, n (%)			0.329
0	45 (67)	16 (52)	
1	11 (16.5)	8 (26)	
≥2	11 (16.5)	7 (22)	
Vaginal delivery binary			**0.022**
Yes	15 (22)	14 (45)	
No	52 (78)	17 (55)	
Smoking, *N* (%)	3 (9.7%)	14 (20.9%)	0.17

Student’s *t*-test and ANOVA for normally distributed data, Mann–Whitney *U* test for non-parametric data, and Chi-square test for categorical data.

In the non-endometriosis group, 20 (65%) patients underwent surgery by laparoscopy: three (15%) sacrocolpopexies, six (30%) ovarian cystectomies, one (5%) myomectomy, four (20%) hysterectomies, four (20%) bilateral adnexectomies, one (5%) ectopic pregnancy and one (5%) laparoscopic fertility management. Eleven patients underwent surgery (35%) by laparotomy: one (9%) ovarian neoplasm debulking surgery, two (18%) hysterectomies and eight (73%) myomectomies. None of these patients had endometriosis: six (21%) had adenomyosis on final histology.

In the endometriosis group, all the patients (n = 67) underwent surgery by laparoscopy. Distribution of the endometriosis lesions and surgical procedures are summarized in Supplementary Table SI. Endometriosis was confirmed on final histology for all patients. Eleven (16%) women had associated adenomyosis.

### Distribution of AGD measurements according to the groups

The average MRI-AGD measurements and their distribution within the groups are summarized in Table II. In the endometriosis and non-endometriosis groups, mean MRI-AGD-AF measurements were 13.3 mm (±3.9) and 21.2 mm (±5.4) (*P* < 10^−5^), respectively. In the endometriosis and non-endometriosis groups, mean MRI-AGD-AC measurements were 40.4 mm (7.3) and 51.1 mm (8.6) (*P* < 10^−5^), respectively.

**Table II:** Evaluation of MRI-AGD according to histological findings.

Groups and *P*-values	MRI-AGD-AF (mm) (SD)	MRI-AGD-AC (mm) (SD)
All patients		
Endometriosis group (n = 67)	13.3 (3.9)	40.4 (7.3)
Non-endometriosis group (n = 31)	21.2 (5.4)	51.1 (8.6)
*P*-value	**<10^−5^**	**<10^−5^**
In patients with prior vaginal delivery		
Endometriosis group (n = 15)	13.5 (4.2)	42.5 (6.9)
Non-endometriosis group (n = 14)	22.6 (5.9)	54.1 (9.5)
*P*-value	**<10^−5^**	**0.0008**
In patients without prior pregnancies		
Endometriosis group (n = 38)	12.9 (3.7)	49 (9.2)
Non-endometriosis group (n = 9)	20.7 (6.4)	39.2 (7.5)
*P*-value	**<10^−5^**	**0.0016**
In obese patients (BMI ≥ 30 kg⋅m^−2^)		
Endometriosis group (n = 12)	13.8 (3.8)	45 (8.6)
Non-endometriosis group (*n* = 10)	21.6 (6.7)	55.4 (10.3)
*P*-value	**0.0025**	**0.018**
Endometriosis group (n = 67)		
Patient with adenomyosis (n = 11)	13.5 (3.7)	41.9 (6.7)
Patient without adenomyosis (n = 56)	13.3 (3.9)	40.1 (7.4)
*P*-value	0.87	0.46

MRI-AGD-AC, anogenital distance from the posterior wall of the clitoris to the anterior edge of the anal canal; MRI-AGD-AF, anogenital distance and from the posterior wall of the vagina to the anterior edge of the anal canal.

Student’s *t*-test.

In the endometriosis group, MRI-AGD-AF and MRI-AGD-AC measurements in patients with and without associated adenomyosis were 13.5 mm (±3.7) and 13.3 (±3.9) (*P* = 0.87), and 41.9 (±6.7) and 40.1 (±7.4) (*P* = 0.46), respectively. The non-endometriosis group did not have enough patients with adenomyosis (six patients) to compare the values between patients with and without adenomyosis.

### Distribution of MRI-AGD according to the endometriosis phenotype and severity

In the endometriosis group, no difference in the average MRI-AGD-AF and MRI-AGD-AC was found between patients with and without endometrioma: 14.5 mm (±4) and 12.6 mm (±3.3) (*P* = 0.97), and 39.5 mm (±6) and 41 mm (±8) (*P* = 0.21), respectively. Similarly, no difference in the average MRI-AGD-AF and MRI-AGD-AC was found between patients with and without bowel endometriosis: 12.9 mm (±3.6) and 13.8 mm (±4.2) (*P* = 0.19) and 39.9 mm (±6.7) and 41.0 mm (±8.0) (*P* = 0.26), respectively.

MRI-AGD-AF and MRI-AGD-AC were not statistically different between patients with a revised score of the American Society of Reproductive Medicine (r-ASRM = stage I, II, III and IV (ANOVA test: *P* = 0.19 and 0.25, respectively). Similarly, MRI-AGD-AF and MRI-AGD-AC were not statistically different between patients with an Enzian score I, II and III (ANOVA test: *P* = 0.94 and 0.92, respectively).

### Univariable and multivariable analysis

Results for the univariable and multivariable linear regression on the MRI-AGD-AF and MRI-AGD-AC are presented in Tables III and IV, respectively. The diagnosis of endometriosis was negatively associated with both the MRI-AGD-AF (*β* = −7.79, 95% CI (−9.88; −5.71), *P* < 0.001) and MRI-AGD-AC (*β* = −9.51 mm, 95% CI (−12.7; 6.24), *P* < 0.001) in multivariable analysis. Age (*β* = +0.31 mm, 95% CI (0.09; 0.53), *P* = 0.006) and BMI (*β* = +0.44 mm, 95% CI (0.17; 0.72), *P* = 0.001) were positively associated with the MRI-AGD-AC measurements in multivariable analysis.

**Table III:** Univariable and multivariable linear regression on the MRI-AGD-AF.

	Univariable		Multivariable	
Variable (SD)	Coefficient 95% CI	*P*-value	Coefficient 95% CI	*P*-value
Age (years)	0.19 (0.045; 0.32)	0.009	0.08 (−0.05; 0.22)	0.24
BMI (kg⋅m^−2^)	0.20 (−0.019; 0.41)	0.07	0.11 (−0.07; 0.28)	0.23
Vaginal delivery (binary)	3.01 (0.56; 5.46)	0.017	0.84 (−2.5; 4.22)	0.62
Parity				
0				
1	5.64 (0.96; 10.31)	0.019	−0.03 (−3.4; 3.4)	0.98
≥2	3.23 (−1.54; 8.0)	0.18		
Endometriosis	−7.88 (−9.78; −5.98)	<1.10^−3^	−7.79 (−9.88; −5.71)	**<1.10^−3^**
Severity of endometriosis				
r-ASRM				
I–II	Reference			
III	2.0 (−0.98; 5.02)	0.184		
IV	1.4 (−0.9; 3.78)	0.226		
Enzian				
1	Reference			
2	−0.2 (−3.41; 3.01)	0.98		
3	−0.43 (−3.2; 2.33)	0.70		

r-ASRM, revised American Society of Reproductive Medicine score.

Simple and multiple linear regression models were used.

**Table IV:** Univariable and multivariable linear regression on the MRI-AGD-AC.

	Univariable		Multivariable	
Variable (SD)	Coefficient 95% CI	*P*-value	Coefficient 95% CI	*P*-value
Age (years)	0.47 (0.26; 0.68)	<1.10^−3^	0.31 (0.09; 0.53)	**0.006**
BMI (kg⋅m^−2^)	0.64 (0.32; 0.97)	<1.10^−3^	0.44 (0.17; 0.72)	**0.001**
Vaginal delivery (binary)	6.18 (2.33; 10.02)	0.002	1.90 (−3.4; 7.21)	0.48
Parity				
0				
1	5.63 (0.96; 10.31)	0.02	0.30 (−5.1; 5.70)	0.911
≥2	3.34 (−1.54; 8.0)	0.18		
Endometriosis	−10.7 (−14.02; −7.36)	<1.10^−3^	−9.51 (−12.7; −6.24)	**<1.10^−3^**
Severity of endometriosis				
r-ASRM				
I–II	Reference			
III	0.83 (−4.8; 6.49)	0.770		
IV	2.66 (−1.7; 7.08)	0.235		
Enzian				
1	Reference			
2	−0.43 (−6.47; 5.6)	0.91		
3	−0.96 (−6.2; 4.25)	0.76		

Simple and multiple linear regression models were used.

We found no correlation between MRI-AGD-AF, MRI-AGD-AC and severity of endometriosis using either r-ASRM or Enzian scores.

### Diagnostic relevance of MRI-AGD and cut-off

**Figure 3.**
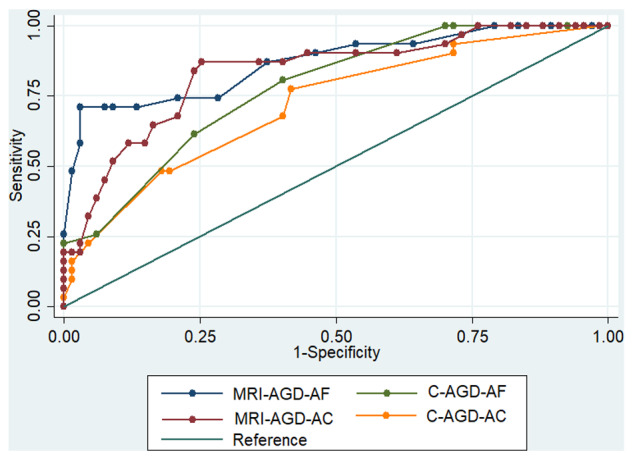
**ROC curves of AGD measures.** C-AGD-AF: green; C-AGD-AC: yellow; MRI-AGD-AF: blue; MRI-AGD-AC: red.

The diagnostic relevance of the MRI measurement of MRI-AGD is represented by the ROC curves in Fig. 3. MRI-AGD-AF and MRI-AGD-AC had an AUC of, respectively, 0.869 (95% CI (0.79; 0.95)) and 0.833 (95% CI (0.746; 0.920)) with no difference between MRI-AGD-AF and MRI-AGD-AC (*P* = 0.35).

**Figure 4.**
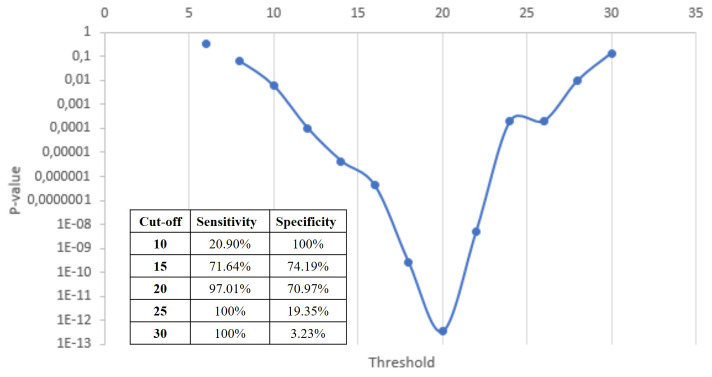
Definition of an optimal cut-off for MRI-AGD-AF.

The definition of an optimal cut-off denoting the strongest correlation between MRI-AGD-AF and the presence of endometriosis selected with a minimum *P*-value approach is summarized Fig. 4. With a cut-off of 20 mm, we obtained a sensitivity of 97.01% and a specificity of 70.97%.

### Comparison between MRI and clinical measurements AGD

ROC curves of MRI-AGD-AF, MRI-AGD-AC, C-AGD-AF and C-AGD-AC are represented in Fig. 3. C-AGD-AF and C-AGD-AC had an AUC of respectively 0.777 (95% CI (0.687; 0.867)) and 0.719 (95% CI (0.612; 0.826)). MRI-AGD-AF was more relevant for the diagnosis of endometriosis than C-AGD-AC (*P* = 0.026). There was no statistical difference between MRI-AGD-AF and C-AGD-AF (*P* = 0.08).

## Discussion

In the present study, surgically and histologically proven endometriosis was associated with a shorter MRI-AGD, especially MRI-AGD-AF, in comparison with patients without endometriosis. No relation was found between MRI-AGD and the severity of endometriosis.

To the best of our knowledge, this is the first study to use MRI to measure AGD in this setting and suggests that MRI-AGD could be a non-invasive tool to diagnose patients with endometriosis. Although MRI-AGD-AF had a better AUC, no difference in the accuracy of endometriosis diagnosis was found between MRI-AGD-AF and MRI-AGD-AC. Moreover, MRI-AGD was independent of the r-ASRM and Enzian classifications, suggesting that this tool could be relevant even in patients with early stages of the disease as observed in the current study. Our data corroborate those of previous studies (Mendiola *et al.*, 2016; Sánchez-Ferrer *et al.*, 2017, 2019) suggesting that clinical AGD evaluation is an appropriate tool for diagnosing endometriosis. However, these studies had several biases related to endometriosis assessment, which was mainly based on symptoms and TVUS without surgical or histological confirmation. For example, Zondervan *et al.* (2020) underlined the non-specific symptomatology of patients with endometriosis that can be attributed to other conditions. Moreover, symptom-based algorithms are also inadequately predictive, as is clinical examination or imaging which has low sensitivity for peritoneal endometriosis (Bazot *et al.*, 2009). However, our previous study on clinical AGD evaluation in patients with histologically proven endometriosis illustrated its relevance even in early-stage disease (Crestani *et al.*, 2020). The optimal MRI-AGD-AF cut-off of 20 mm used to distinguish patients with and without endometriosis was identical to the clinical optimal cut-off (Crestani *et al.*, 2020).

Laparoscopy is currently the gold standard for the diagnosis of superficial endometriosis. However, it is an invasive technique exposing the patients to surgical complications just for a potential diagnosis (Khan *et al.*, 2018). Bazot *et al.* (2009) reported that pelvic MRI had an accuracy of 57%, sensitivity of 5% and specificity of 94% for the diagnosis of superficial endometriosis. More recently, Thomeer *et al.* (2014) reported a sensitivity of 100% for MRI for the diagnosis of r-ASRM stages II, III and IV endometriosis, but only 42% for stage I (Berger *et al.*, 2018; Khan *et al.*, 2018). The AGD measurement may thus be particularly relevant for these patients as we found that MRI-AGD measurements were not correlated to the severity of the disease: MRI-AGD measures were similar regardless of the ASRM score, ENZIAN score, presence of digestive endometriosis, and endometrioma. This finding is in accordance with our previous results on C-AGD measurements in patients with endometriosis (Crestani *et al.*, 2020). However, our expert centre mainly manages complex cases of endometriosis as confirmed by the high proportion of patients with colorectal endometriosis in our study population. In the present cohort, certain subgroups contain a relatively low number of patients, especially women with minimal or mild stages of endometriosis. Therefore, we cannot exclude that slight differences in AGD measurements depending on the endometriosis severity were missed, due to a lack of power.

The performance of pelvic MRI for the diagnosis of endometriosis is correlated with the radiologist’s experience, even for DE, hence the images should ideally be interpreted in an expert centre (Chanavaz-Lacheray *et al.*, 2018; Thomassin-Naggara *et al.*, 2018; Bruyere *et al.*, 2020). However, in women undergoing exploration for chronic pelvic pain, most routine pelvic MRIs are interpreted by radiologists with a limited experience in endometriosis. In this specific setting, MRI-AGD-AF is an easily assessable finding that does not require specific knowledge of endometriosis imaging: it merely requires identifying the posterior fourchette to the anterior wall of the anal canal. In the present study, using an optimal cut-off of 20 mm, MRI-AGD-AF was able to diagnose endometriosis with a sensitivity of 97% and a specificity of 71%. Moreover, we showed that MRI-AGD-AF had an AUC of 0.869 (95% CI (0.79; 0.95)).

Several limitations of our study deserve to be highlighted. First, it was a retrospective analysis on a specific population, managed in an expert centre in endometriosis. As previously mentioned, most of the included patients had severe diseases and the number of patients with ASRM stages I and II was rather small. Consequently, an overestimation of the diagnostic test performances, caused by a spectrum bias, is possible. However, either in our data or in the literature, there is no evidence of a correlation between AGD measurements and the severity of endometriosis. Second, the MRI-AGD measurement was performed by an expert radiologist blinded to surgical findings but not to the entire pelvic examination. As most patients had a severe form of endometriosis, visible lesions could have biased the measurement. However, as precise anatomical landmarks were defined, we do not believe that this will have altered the results. Third, the MRI-AGD measurements differed from C-AGD. On average, MRI-AGD-AF and MRI-AGD-AC were, respectively, 9 mm (±7) and 44 mm (±16) shorter than C-AGD-AF and C-AGD-AC. This difference is explained by the technique of measurement. MRI-AGD was the shorter direct line between two landmarks on a sagittal plane, whereas C-AGD involves the curve of the vulva. This could also explain the higher C-AGD values in patients after vaginal delivery. Moreover, the chosen upper and lower landmarks were different: respectively, the clitoral surface and anus for C-AGD, and the anterior vaginal wall and anterior edge of the anal canal for MRI-AGD. Fourth, our findings remain to be validated among adolescents who were not included in this study and for whom MRI-AGD could be particularly relevant. Finally, some patients belonging to the control group had diseases, which may imply a sub-optimal *in utero* hormonal environment and therefore a bias in the results. In any case, as we had chosen a surgical and histological gold standard for the diagnosis of endometriosis, this bias could not be avoided.

In conclusion, this study strengthens the argument in favour of the AGD as an anatomical marker of endometriosis. Clinical and radiological AGD measurements may be used as diagnostic tools for this disease. Our data support the introduction of MRI-AGD-AF measurement as part of the exploration of patients with suspected endometriosis, undergoing an MRI examination.

## Supplementary data

Supplementary data are available at *Human Reproduction Open* online.

## Authors’ roles

Study concept and design: A.A. and E.D. Acquisition of data: A.C., M.B. Statistical analysis: C.F. and S.B. Interpretation and synthesis of data: A.A., S.P., K.K. and E.D. Drafting the manuscript: A.C. Supervision and critical revision of the manuscript for important intellectual content: A.A., C.F., E.D. and S.B. All authors have read, and confirm that they meet, the authorship criteria.

## Funding

None.

## Conflict of interest

None.

## Supplementary Material

hoab003_Supplementary_Data
