# Supplementary material for: A short anogenital distance on MRI is a marker of endometriosis
Source: Hum Reprod Open. 2021 Feb 17;2021(1):hoab003. doi: 10.1093/hropen/hoab003 (PMC7887775; doi:10.1093/hropen/hoab003)
Supplement: hoab003_Supplementary_Data [file hoab003_supplementary_data.docx]

| Characteristics | Number N (%) of patients in the endometriosis group |
| --- | --- |
| **Distribution of endometriosis lesions**  Endometrioma and deep endometriosis  Endometrioma alone  Torus uterinum  Utero-sacral ligament endometriosis  Vaginal endometriosis  Bladder endometriosis  Adenomyosis  Colorectal endometriosis | 25 (37)  1 (1)  55 (82)  54 (81)  17 (25)  2 (3)  11 (16)  37(55) |
| **Distribution of patients according to r-ASRM and Enzian scores**  **r-ASRM**  r-ASRM I  r-ASRM II  r-ASRM III  r-ASRM IV  **Enzian**  Grade 1  Grade 2  Grade 3 | 5 (7)  10 (15)  12 (18)  40 (60)  10 (15)  15 (22)  42 (63) |
| **Type of colorectal surgery**  Shaving  Discoid excision  Segmental resection | 10 (15)  10 (15)  17 (25) |

**Supplementary** **Table SI** Distribution of endometriosis lesions and surgical procedures in 67 women.

r-ASRM revised American Society of Reproductive Medicine score; DE deep endometriosis

Some patients had multiple synchronous locations.
